# Hathewaya limosa Empyema: A Case Report

**DOI:** 10.7759/cureus.55156

**Published:** 2024-02-28

**Authors:** Pradeep Kumar Mada, Muhammad H Khan

**Affiliations:** 1 Infectious Diseases, Comanche County Memorial Hospital, Lawton, USA; 2 Surgery, Michigan State University, East Lansing, USA

**Keywords:** total lung decortication, anaerobic microorganisms, clostridium limosum, pleural empyema, hathewaya limosa

## Abstract

*Hathewaya limosa*, an anaerobic bacterium, has been associated with various infections, including prosthetic valve endocarditis, although its role in empyema remains uncommon. This abstract presents a case report of a patient diagnosed with *H. limosa *empyema, highlighting the clinical presentation, diagnostic challenges, and successful treatment strategies. The case underscores the importance of considering unusual pathogens in the context of empyema. We discuss the clinical management, microbiological identification, and outcomes of this rare infection to contribute valuable insights for healthcare practitioners encountering similar cases.

## Introduction

Pleural empyema is defined as the presence of pus, a positive culture, or Gram stain, in addition to features of complicated effusion. The common clinical features include a productive cough, fever, pleuritic chest pain, and dyspnea. It requires drainage plus decortication or video-assisted thoracoscopic surgery (VATS) for loculated effusions with extensive pleural fibrosis. The common pathogens implicated in the causation of acute pleural empyema are *Streptococcus pneumoniae*, Streptococcus sp. (group A), *Staphylococcus aureus* (MSSA and MRSA), and *Hemophilus influenzae* [1]. A new genus, Hathewaya genus novom (gen. nov.), was recommended for the species *Clostridium histolyticum*, *C. limosum*, and *C. proteolyticum* as *Hathewaya histolytica* genus novum combinatio nova (comb. nov.), *Hathewaya limosa* comb. nov. and *H. proteolytica* comb. nov. [2]. *H. limosa* is an anaerobic, Gram-positive, spore-forming bacillus that typically inhabits the soil and has been isolated from infections in various animals, such as chickens, alligators, and cattle. Animal studies have shown that it causes muscle and tissue damage through the action of collagenases and lecithinases [3]. To date, there has been only one reported human infection caused solely by *H. limosa*, observed in a case of prosthetic valve endocarditis [4]. We present a case of *H. limosa* pleural empyema, which was treated with combined surgical intervention and intravenous antibiotics with a successful outcome.

## Case presentation

**Figure 1:**
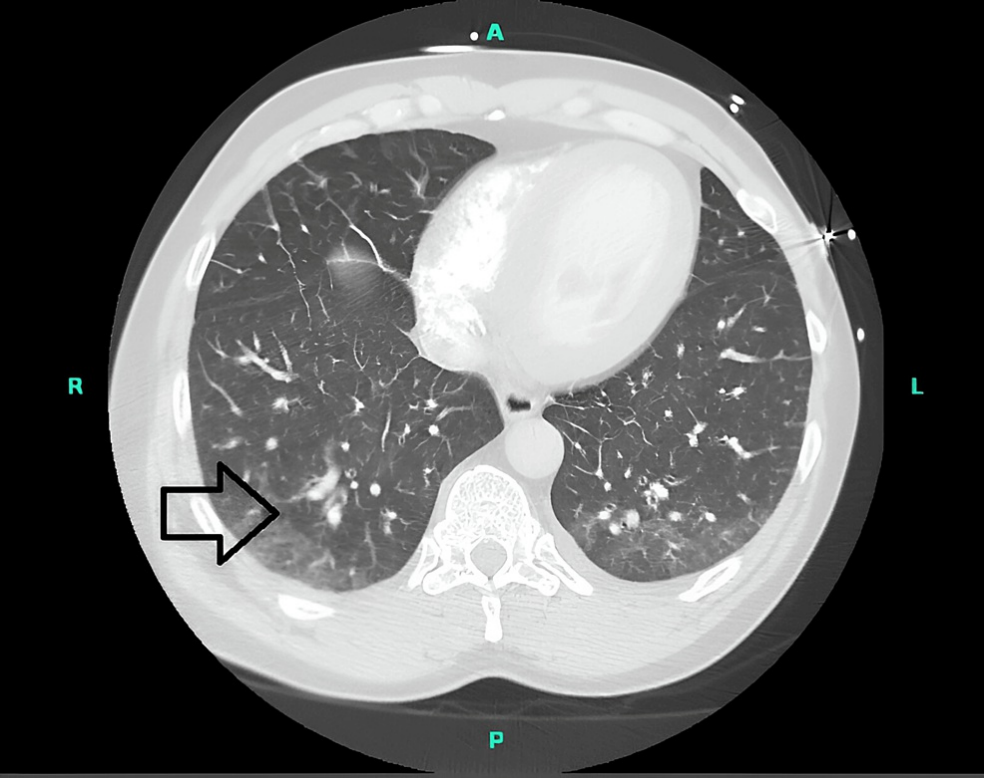
CT chest on admission CT showed mild alveolar opacities concerning pulmonary edema or pneumonia and emphysema (black arrow)

A 59-year-old male with a history of essential hypertension and cigarette smoking (1 pack/day for 35 years) presented with shortness of breath. The patient reported that he had progressive shortness of breath for one week, associated with a nonproductive cough. He denied sick contacts, surgeries, trauma, dental procedures, or recent travel. He was afebrile and hemodynamically stable on admission. The chest X-ray showed no acute cardiopulmonary process. Initial ABG showed a pH of 6.95, a pO_2_ of 74 millimetres of mercury (mmHg), and a pCO_2_ of 70 mmHg. He was intubated and put on a mechanical ventilator. On day 1, a computed tomography (CT) scan of the chest showed mild alveolar opacities concerning pulmonary oedema, pneumonia, and emphysema (Figure 1).

**Figure 2:**
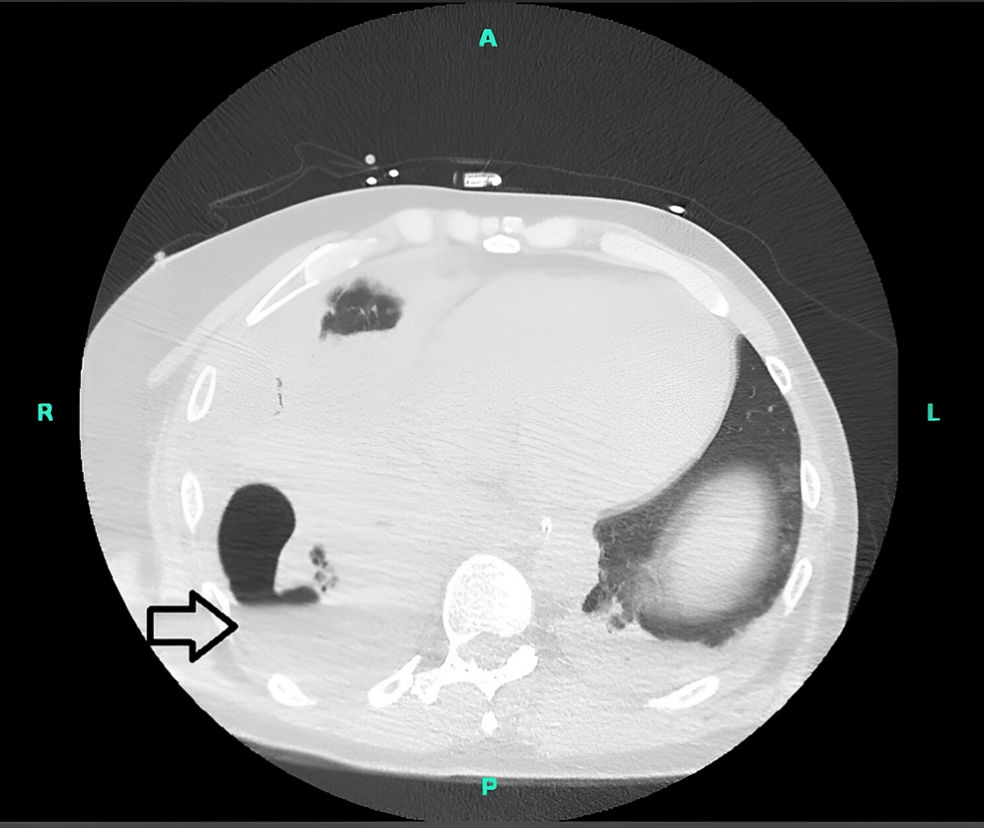
CT chest on day 4 of hospital admission revealed right-sided empyema thoracis (black arrow)

Human immunodeficiency virus (HIV) screen, nasal swab for coronavirus disease (COVID-19), influenza, and respiratory syncytial virus (RSV) were negative. Sputum cultures grew a heavy growth of *Haemophilus influenzae* (beta-lactamase-negative). He was started on IV ceftriaxone (2 g) once daily. On day 4, the patient spiked a temperature to 101 °F. An X-ray of the chest showed right-sided lower lobe consolidation. Repeat CT chests revealed bilateral airspace consolidations, most consistent with multifocal pneumonia and right-sided loculated fluid collection (Figure 2).

Blood cultures remained negative for any growth. Due to the concern of right empyema thoracis, he underwent right posterior lateral serratus sparing thoracotomy, right total lung decortication, and chest tube placement. During surgery, immediately upon entering the hemithorax, a large amount of brownish, purulent fluid with a distinct foul and fetid odor was identified. Operative findings also included dense fibrinopurulent empyema on the right side, destruction of the lateral aspect of the right lower lobe, and extensive cortical peeling over the right upper lobe, right middle lobe, and right lower lobe. Surgical cultures grew heavy growth of *H. limosa* and Bacteroides fragilis, identified by matrix-assisted desorption/ionization time-of-flight mass spectrometry (MALDI-TOF MS). Antibiotic sensitivity testing for anaerobes was not available at our facility. Pathological examination revealed dense fibrinopurulent material, necrosis, and scattered pigment-laden histiocytes, and no evidence of malignancy was identified. He was discharged on four weeks of intravenous metronidazole 500 mg four times a day. The patient demonstrated a favorable response to the combined surgical and medical treatment, with a resolution of symptoms and radiological improvement observed during follow-up. A follow-up CT chest after four months reported evidence of post-inflammatory/post-infectious remnants or developing scar development in the posterior lateral right lower lobe, with a resolution of the previous right complex pleural effusion.

## Discussion

Similar to our case, a few case reports have illustrated *H. limosa* coexistence with other bacterial and fungal organisms, contributing to various cardiac, gastrointestinal, and dermatological infections in patients with hypertension and diabetes mellitus [5-7]. To the best of our knowledge, this is the first reported case of empyema attributable to *H. limosa*. In our case, the patient, a tobacco user, had a history of hypertension and was admitted with shortness of breath from community-acquired pneumonia from *Hemophilus influenzae*. After four days of intubation, he developed a right-sided empyema. While Clostridia species have been implicated in pleuro-pulmonary disease [8-12], it is uncommon to have pulmonary infections without preceding surgical treatments or trauma [13]. Along with the isolation of Hathewaya limosa from the pleural fluid, Bacteroides, another group of gram-negative rods ubiquitous in the human gut, were identified [14]. This finding is consistent with two previous case reports that documented the coexistence of other organisms with H. limosa [5,7]. Previous case series have indicated that Clostridial pleuro-pulmonary infections can occur due to aspiration of oropharyngeal contents, invasive procedures involving the pleural cavity, and hematogenous routes [15]. Our patient was intubated, and aspiration seems to be a more plausible mechanism of entry into the pleural space. In addition, the patient’s history of tobacco use and emphysema may have made him susceptible to the infection.

The suspicion for anaerobic infection rises when a wound has a foul smell or when a Gram stain of pus from an infected site shows mixed pleomorphic bacteria but aerobic cultures show no growth. Mixed anaerobic infections arise when the usual commensal relationship among the normal flora of mucosal surfaces is disrupted by surgery, injury, or ischemia. Since aerobic and anaerobic bacteria are often present at the same infected site, aerobic and anaerobic cultures for isolation are necessary to not miss anaerobes. The overall mortality rate for mixed anaerobic pneumonias tends to be high [16]. The management of empyema typically involves a combination of antibiotics and surgical interventions [17]. In our patient, total lung decortication and a course of IV metronidazole were pivotal in improving the clinical condition. While we had success utilizing only metronidazole, antibiotic sensitivity testing should be done when available.

The antibiotic regimen we selected aligns with the current literature that has demonstrated Clostridium species to be highly susceptible to metronidazole, meropenem, and piperacillin [18]. However, there have been reports of reduced susceptibility to metronidazole, while resistance to other antimicrobials, such as erythromycin and moxifloxacin, demonstrates variability across regions [19,20]. These findings highlight the importance of testing antibiotic susceptibility to the importance of monitoring resistance, and providing appropriate region-based therapy for *H. limosa* infections.

## Conclusions

*Hathewaya limosa* empyema is a rare clinical entity that requires surgical evacuation and antibiotic treatment. Timely diagnosis, appropriate antimicrobial therapy, and surgical intervention contributed to a successful outcome in this case.
